# Serum MicroRNA Profiles Serve as Novel Biomarkers for the Diagnosis of Alzheimer's Disease

**DOI:** 10.1155/2015/625659

**Published:** 2015-05-20

**Authors:** Hui Dong, Jialu Li, Lei Huang, Xi Chen, Donghai Li, Tao Wang, Caiyou Hu, Jun Xu, Chunni Zhang, Ke Zen, Shifu Xiao, Qiao Yan, Cheng Wang, Chen-Yu Zhang

**Affiliations:** ^1^Jiangsu Engineering Research Center for MicroRNA Biology and Biotechnology, State Key Laboratory of Pharmaceutical Biotechnology, School of Life Sciences, Nanjing University, Nanjing 210093, China; ^2^Department of Clinical Laboratory, Jinling Hospital, Nanjing University School of Medicine, Nanjing 210002, China; ^3^Key Laboratory of Cancer Prevention and Therapy, Tianjin Medical University Cancer Institute and Hospital, Tianjin 300060, China; ^4^Department of Geriatric Psychiatry, Shanghai Mental Health Center, Shanghai Jiaotong University School of Medicine, Shanghai 200030, China; ^5^Alzheimer's Disease and Related Disorders Center, Shanghai Jiaotong University, Shanghai 200000, China; ^6^Department of Neurology, Jiangbin Hospital, Guangxi Zhuang Autonomous Region, Nanning 530021, China; ^7^Department of Neurology, Nanjing Brain Hospital, Nanjing 210029, China

## Abstract

Alzheimer's disease (AD) is the most common type of dementia, and promptly diagnosis of AD is crucial for delaying the development of disease and improving patient quality of life. However, AD detection, particularly in the early stages, remains a substantial challenge due to the lack of specific biomarkers. The present study was undertaken to identify and validate the potential of circulating miRNAs as novel biomarkers for AD. Solexa sequencing was employed to screen the expression profile of serum miRNAs in AD and controls. RT-qPCR was used to confirm the altered miRNAs at the individual level. Moreover, candidate miRNAs were examined in the serum samples of patients with mild cognitive impairment (MCI) and vascular dementia (VD). The results showed that four miRNAs (miR-31, miR-93, miR-143, and miR-146a) were markedly decreased in AD patients' serum compared with controls. Receiver operating characteristic curve analysis demonstrated that this panel of four miRNAs could be used as potential biomarker for AD. Furthermore, miR-93, and miR-146a were significantly elevated in MCI compared with controls, and the panel of miR-31, miR-93 and miR-146a can be used to discriminate AD from VD. We established a panel of four serum miRNAs as a novel noninvasive biomarker for AD diagnosis.

## 1. Introduction

Alzheimer's disease (AD) is a neurodegenerative disease that is characterized by progressive memory loss and behavioral changes. In 2006, the worldwide prevalence of AD was 26.6 million, and by 2050, AD is predicted to affect 1 in 85 people globally [1]. Currently, the clinical diagnosis of AD is largely based on the criteria established by the National Institute of Neurological Disorders and Stroke in collaboration with the Alzheimer's Association (NINDS-AA) [2]. However, the specificity of diagnosing probable AD using these criteria is only approximately 70% [3]. These criteria are based on patient history, psychological testing, brain imaging, and the exclusion of other neurological disorders. To make a diagnosis based on these criteria is complicated and is often influenced by subjective factors such as a patient's intelligence. Generally, the disease is thought to develop in a preclinical stage for many years before progressing into typical AD. Using the current clinical methods, AD is not detectable in these patients. It is crucial that the disease is diagnosed as early as possible so that early interventions, such as cholinesterase inhibitors, episodic memory trainings, cognitive trainings, and combined attention and memory training, can be deployed to slow disease progress [4]. Therefore, it is now urgent to develop simple, specific, and noninvasive biomarkers that can help in diagnosing AD in time to inhibit its development.

Mild cognitive impairment (MCI) patients have mild impairments in some domains of cognition but have no symptoms of dementia. However, most of these patients will go on to develop AD [5]. Thus, identifying MCI is crucial for delaying its development into AD. Similar to AD, vascular dementia (VD) also involves cognitive impairment; however, unlike AD, the neurodegeneration observed in VD is due to pathological lesions caused by vascular ischemia or occlusion [6]. Additionally, VD is the second most common type of dementia after AD in the United States [7]. Differentiating these two forms of dementia (AD and VD), which are often confused in clinical practice, is important both in theory and in the clinic.

MicroRNAs (miRNAs) are a type of small noncoding RNA that act as endogenous regulators of gene expression by binding to complementary sequences of target messenger RNA (mRNA). The dysregulation of miRNAs is thought to be involved in many diseases [8]. In a recent study, we found that human serum contains a considerable number of stable miRNAs [9]. Other studies have also demonstrated that the expression profile of serum miRNAs could serve as a novel biomarker for the diagnosis of many other diseases such as diffuse large B cell lymphoma and muscular dystrophy [10–12]. Dysregulated serum miRNA events, such as the downregulation of miR-137, miR-181c, miR-9, and miR-29a/b in the blood of AD patients, were reported [13]. A number of dysregulated plasma miRNAs have also very recently been identified [14–16]. However, due to the small sample sizes in the above reports, there is a lack of an extensive and genome-wide analysis for the diagnostic value of miRNAs in AD patients.

In this study, we used Solexa sequencing and hydrolysis probe-based reverse transcription quantitative real-time PCR (RT-qPCR) to detect the expression profile of miRNAs in the serum of AD patients. The results demonstrated that the expression profile of four serum miRNAs, including miR-31, miR-93, miR-143, and miR-146a, can serve as simple, specific, and noninvasive biomarkers that can help in diagnosing Alzheimer's disease.

## 2. Materials and Methods

### 2.1. Participants

**Figure 1:**
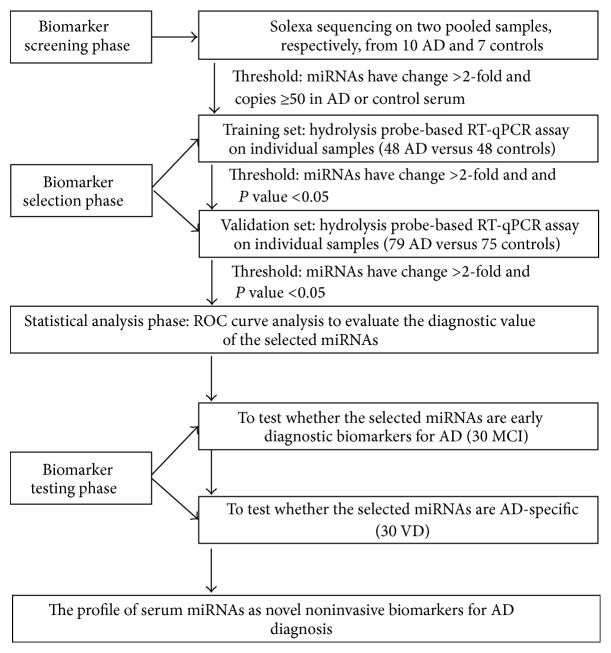
An overview of the experimental design strategy.

The present study enrolled 127 AD patients, 30 MCI patients, and 30 VD patients. All of the patients underwent a detailed clinical examination and were diagnosed using the NINDS-AA criteria and laboratory evaluation as nondementia controls or as having MCI, AD, or VD. The demographics and clinical features of the patients are listed in Table 1. A multiphase study was designed to identify serum miRNAs as surrogate biomarkers for AD (Figure 1). In the initial biomarker screening stage, Solexa sequencing was used to identify miRNAs that were markedly different from the AD cases and age-matched nondementia controls. The differentially expressed serum miRNAs identified in the Solexa survey were subsequently examined via a two-phase experimental procedure using hydrolysis probe-based RT-qPCR. In the biomarker selection phase, serum samples from 48 AD patients who were treated at the Shanghai Mental Health Center, Nanjing Brain Hospital, and Guangxi Jiangbin Hospital formed the training set. Additionally, in the biomarker validation phase, serum samples from additional 79 AD patients from the Shanghai Mental Health Center, Nanjing Brain Hospital, or Guangxi Jiangbin Hospital formed the validation set. Samples from 30 MCI patients and 30 VD patients were also obtained from the Shanghai Mental Health Center. Additionally, 123 individuals who were recruited from the Nanjing Brain Hospital, Guangxi Jiangbin Hospital, and Shanghai Mental Health Center and showed no evidence of dementia disease were selected as nondementia controls.

Written informed consent was obtained from all patients and nondementia participants prior to the study. For the patients who had a compromised capacity to consent, written informed consent was obtained from the legally authorized representative on behalf of the participant. The study protocol was approved by the ethics committees of Shanghai Mental Health Center, Nanjing Brain Hospital, and Guangxi Jiangbin Hospital. The study was performed in accordance with the 1975 Declaration of Helsinki and the REMARK guidelines for biomarker studies.

### 2.2. Sample Processing and RNA Extraction

For the Solexa sequencing assay, we pooled the sera from 10 AD patients (approximately 60 mL) for case sample pools and the sera from 7 nondementia controls (approximately 60 mL) for control sample pools. Then, we isolated the total RNA from each pooled sera sample using TRIzol reagent (Invitrogen) according to the manufacturer's instructions with minor modifications. The RNA pellet was collected from the aqueous phase via three steps of acid phenol-chloroform purification. Finally, the pellet was dissolved in 30 *μ*L of DEPC water and stored at −80°C until further analysis. For the RT-qPCR assay, total RNA was extracted from 100 *μ*L of sera using one-step phenol-chloroform purification [17].

### 2.3. Biomarker Screening Stage

The Solexa sequencing procedure and* in silico* analysis were performed as previously described [9].

### 2.4. Quantification of miRNAs via RT-qPCR

Hydrolysis miRNA probes (Applied Biosystems, Foster City, CA, USA) were used to perform the RT-qPCR according to the manufacturer's instructions. Briefly, 2 *μ*L of total RNA was reverse-transcribed to cDNA using the AMV reverse transcriptase (TaKaRa, Dalian, China) and a stem-loop RT primer (Applied Biosystems). Real-time PCR was performed using a TaqMan PCR kit on an Applied Biosystems 7300 Sequence Detection System (Applied Biosystems). All reactions, including no-template controls, were performed in triplicate. Because traditional reference genes, such as U6 and 5S rRNA, are degraded in serum samples, they cannot be used as an internal control in our system; thus, miRNA expression was normalized to serum volume in the present study. During RT-qPCR analysis, the experimenters were blinded to the AD and control samples, and the AD samples and controls were mixed on the RT-qPCR plates to avoid batch effects. Furthermore, when the expression levels of the target miRNAs were examined, synthetic miRNA oligonucleotides at known concentrations were prepared via a tenfold serial dilution (10^3^ fmol/L~10^6^ fmol/L) and were also reverse-transcribed and amplified. The synthetic miRNA levels were assessed using the RT-qPCR assay. The resulting *C*_*q*_ values were plotted versus the log_10_ of the amount of the synthetic miRNAs to generate calibration curves. The absolute concentrations of the serum miRNAs were calculated using the corresponding calibration curves.

### 2.5. Statistical Analysis

Statistical analysis was performed using SPSS 16.0 software. Data were presented as the means ± SEM for miRNAs or means ± SD for other variables. The nonparametric Mann-Whitney *U*-test was used to compare the concentration differences of the miRNAs between groups. Student's *t*-test or a two-sided *χ* ^2^ test was used to compare the differences in other variables between the two groups. A *P* value < 0.05 was considered statistically significant. A risk score analysis was performed to evaluate the associations between serum miRNA levels and AD. Finally, we presented the receiver operating characteristic (ROC) curve analysis and calculated the area under the curve (AUC) of each miRNA and their panel to evaluate the diagnostic effect of the profiles.

## 3. Results

### 3.1. Solexa Sequencing Analysis

Solexa sequencing was used to determine miRNAs that had significantly altered expression in the AD cases compared with the control subjects. Serum samples from 10 AD patients and from 7 nondementia controls were separately pooled. The total RNA from the two pooled samples was then extracted for Solexa sequencing. The Solexa data revealed that miRNAs were the major components of small RNAs in the serum (see Supplemental Data Table S1 in the Supplementary Material available online at http://dx.doi.org/10.1155/2015/625659). Additionally, 239 and 204 miRNAs were detected in the nondementia controls and AD patients, respectively (see Supplemental Data Table S2). We selected the miRNAs that showed at least a 2-fold change in expression between the patient and control group, as well as those that had more than 50 copies in either group. Based on these criteria, 38 miRNAs were expressed at different levels in AD compared with the controls (see Supplemental Data Table S3). Of the 38 dysregulated miRNAs determined via initial Solexa sequencing, 10 were upregulated and 28 miRNAs were downregulated in the AD group. Fifteen of the dysregulated miRNAs, including 5 upregulated miRNAs and 10 downregulated miRNAs (which have been reported to be conserved in brain tissue or related with cell apoptosis and AD) were then chosen and subjected to additional validation via RT-qPCR. Moreover, miR-148a, which has been reported to be dysregulated in AD and to participate in neural development, was also recruited according to RT-qPCR even though the Solexa sequencing data showed 49 (and not 50) reads of this miRNA in the controls.

### 3.2. Evaluation of miRNA Expression via RT-qPCR Analysis

We next detected the expression levels of the 16 candidate miRNAs selected from the Solexa sequencing using a hydrolysis probe-based RT-qPCR assay. The serum samples were arranged in two independent sets: a training set and a validation set (Figure 1). As shown in Table 1, there were no significant differences regarding age, gender, and years of education between the AD patients and control subjects.

**Figure 2:**
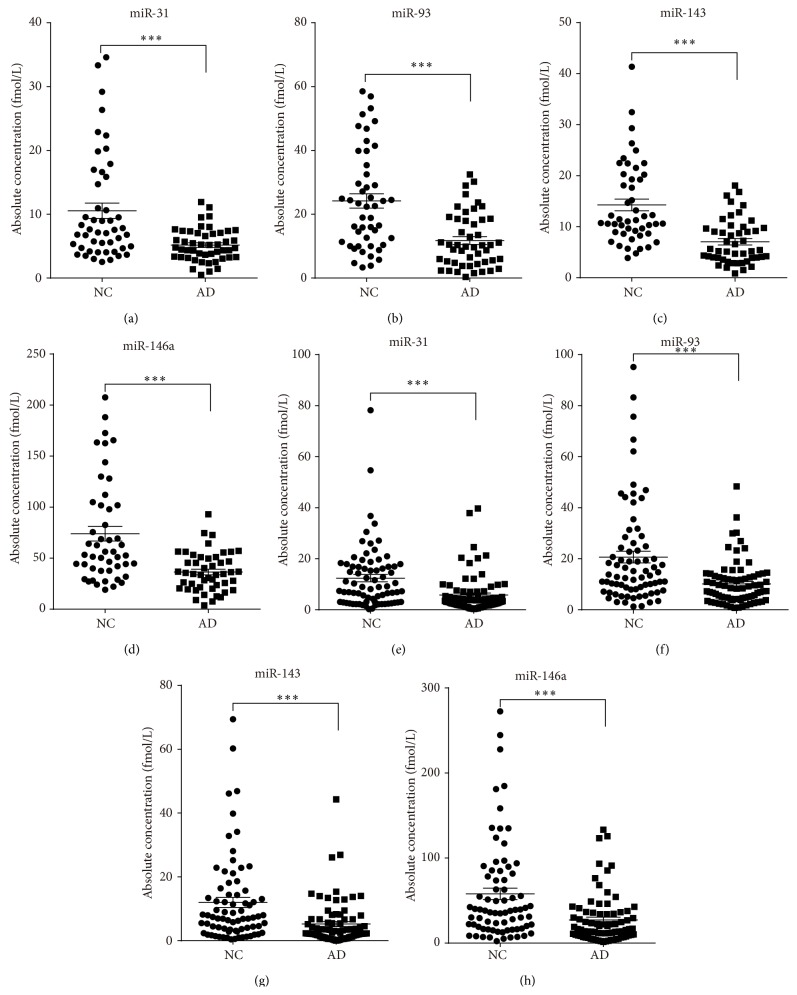
The concentrations of 4 identified serum miRNAs in Alzheimer's disease (AD) cases and nondementia controls (NC) enrolled in the training and validation sets. The absolute concentrations of the 4 identified serum miRNAs in 48 AD and 48 NC of training set (a–d), as well as 79 AD and 75 NC of the validation set using a RT-qPCR assay (e–h). The absolute concentrations of the miRNAs were calculated based on the standard curves. Each point represents the mean of triplicate samples. Each *P* value was derived from a nonparametric Mann-Whitney *U*-test.^*∗∗∗*^ *P* < 0.001.

The 16 miRNAs selected from the Solexa sequencing were first confirmed in the training set (48 AD patients and 48 nondementia controls) using hydrolysis probe-based RT-qPCR. Consequently, 6 of the miRNAs (miR-31, miR-93, miR-143, miR-146a, miR-148a, and miR-191) were found to be significantly decreased in the AD patients (all *P* < 0.05), whereas the other 10 miRNAs (miR-122, miR-23a, miR-103, miR-193a-5p, miR-874, miR-23b, miR-221, miR-199a-3p, miR-7, and miR-885-3p) showed no significant difference between the AD patients and controls (see Supplemental Data Table S4). Of the markedly altered serum miRNAs examined in the training set, a miRNA with a mean fold-change ≥ 2 and *P* value < 0.05 was then selected for further analysis. Moreover, the miRNAs that had a *C*_*q*_ value > 35 and a detection rate < 75% in either the AD or control group were discarded. Based on these criteria, a list of four miRNAs with a differential expression pattern between the AD patients and controls was generated. These miRNAs were miR-31, miR-93, miR-143, and miR-146a (Table 2 and Figure 2).

To verify these four miRNAs as a signature for AD, we further assessed their expression level in a larger independent set (validation set) of serum samples that included 79 AD patients and 75 controls using hydrolysis probe-based RT-qPCR. Consistent with the results of the training set, the serum levels of the four miRNAs were significantly lower in the AD patients compared with the control subjects (Table 2 and Figure 2).

### 3.3. ROC Curve Analysis

**Figure 3:**
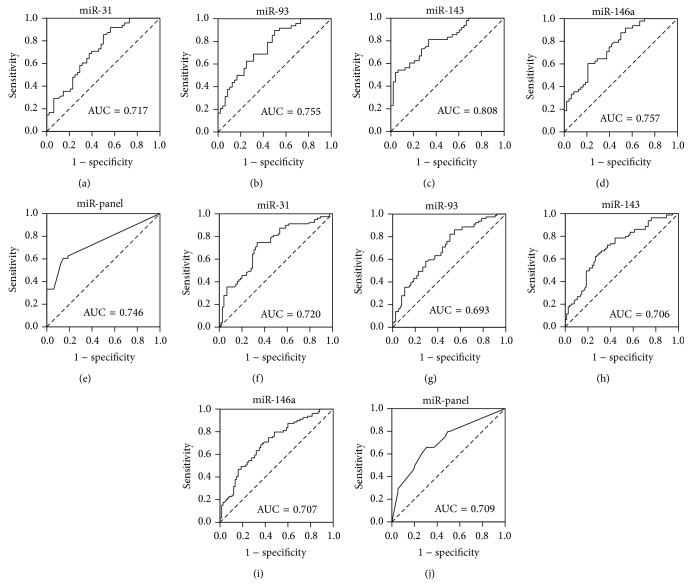
The receiver operating characteristic (ROC) curve analysis for discriminative ability between Alzheimer's disease (AD) cases and nondementia controls (NC) enrolled in the training and validation sets by the 4 miRNAs and their panel. ROC curves for the 4 miRNAs and the 4-miRNA panel to separate 48 AD cases from 48 NC in the training set (a–e) and 79 AD cases from 75 NC in the validation set (f–j).

To evaluate the diagnostic value of the 4 significantly altered miRNAs, a receiver operating characteristic curve (ROC) was created to evaluate the sensitivity and specificity of the four miRNAs as serum biomarkers for AD in the training and validation sets, respectively. The areas under the curve (AUC) of the four miRNAs in the training set were as follows: miR-31, 0.717 (95% CI, 0.615–0.818); miR-93, 0.755 (95% CI, 0.660–0.850); miR-143, 0.808 (95% CI, 0.723–0.892); and miR-146a, 0.757 (95% CI, 0.663–0.851) (Figure 3). For the serum samples in the validation set, the AUC for the four miRNA were as follows: miR-31, 0.720 (95% CI, 0.639–0.801); miR-93, 0.693 (95% CI, 0.611–0.776); miR-143, 0.706 (95% CI, 0.624–0.788); and miR-146a, 0.707 (95% CI, 0.625–0.788) (Figure 3). To further assess the diagnostic usefulness of the selected four serum miRNAs in discriminating between AD and controls, we conducted a risk score analysis to construct a signature using these 4 miRNAs. Each AD patient or control was assigned a risk score based on a linear combination of the levels of the 4 miRNAs weighted by their regression coefficients as previously described [16, 18]. The AUC for the combination of the four miRNAs was 0.746 (95% CI, 0.647–0.846) and 0.709 (95% CI, 0.628–0.791) for AD in the training and in validation sets, respectively (Figure 3). Taken together, these results indicated that the profile of the four serum miRNAs could be a simple, specific, and noninvasive molecular biomarker for diagnosing AD.

### 3.4. Expression Levels of the Four miRNAs in MCI Cases and VD Cases

**Figure 4:**
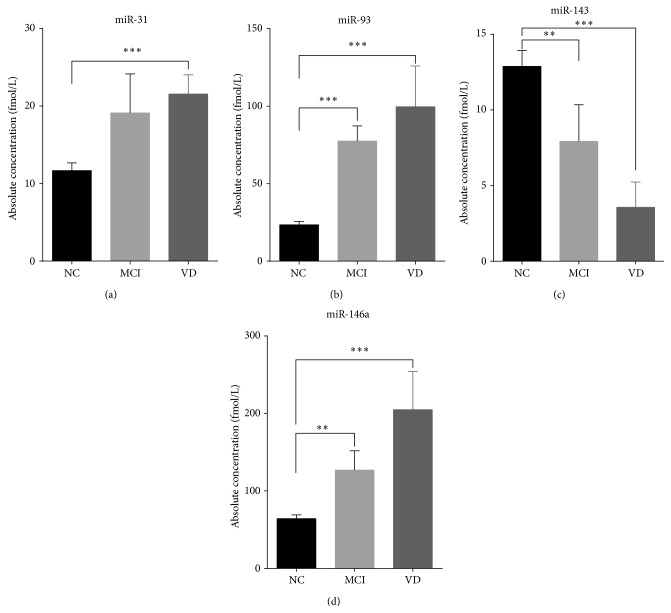
The concentrations of the 4 identified serum miRNAs in mild cognitive impairment (MCI) cases, vascular dementia (VD), and all nondementia controls (NC) enrolled in the study. The absolute concentrations of 4 identified serum miRNAs in 30 MCI, 30 VD, and 123 NC patients using a RT-qPCR assay (a–d). The absolute concentrations of the miRNAs were calculated based on the standard curves. Each *P* value was derived using a nonparametric Mann-Whitney *U*-test. ^*∗∗*^ *P* < 0.01.

To determine whether the four miRNAs could serve as a biomarker to discriminate AD from MCI and other dementia diseases, we next determined the expression levels of the four miRNAs in 30 MCI patients using hydrolysis probe-based RT-qPCR. The results showed that the concentrations of miR-93 and miR-146a were significantly elevated in MCI, whereas miR-143 was significantly deceased in MCI compared with the controls. No obvious difference was found for miR-31 (Figure 4). We then examined the expression levels of the four miRNAs in another 30 VD patients using hydrolysis probe-based RT-qPCR. The concentrations of serum miR-143 were markedly downregulated in the VD patients compared with the controls. Unlike miR-143, the levels of miR-31, miR-93, and miR-146a were significantly higher in the VD patients than in the controls, which indicates that the expression profile of miR-31, miR-93, and miR-146a could potentially serve as a biomarker for AD (Figure 4). Collectively, based on the above results, we suspected that upregulated miR-31, miR-93, and miR-146a combined with decreased miR-143 in serum could delineate MCI and VD from AD.

## 4. Discussion

Currently, the clinical diagnosis of AD primarily depends on patient history, psychological testing, brain imaging, and the exclusion of other neurological disorders. However, this clinical diagnosis method is complicated and expensive. Therefore, simple and convenient biomarkers are urgently needed to help diagnose AD at an early stage. In this study, we employed Solexa sequencing of pooled sera samples followed by multiple RT-qPCR verification assays of individual samples to generate disease-associated serum miRNA profiles. This paper presents one of the most systematic examinations and genome-scale descriptions of miRNAs in the serum of AD patients and demonstrates that the expression profile of four serum miRNAs, including miR-31, miR-93, miR-143, and miR-146a, can serve as a noninvasive biomarker for AD diagnosis.

Several studies recently reported aberrant miRNA expression in AD brains [19–25]. These results indicated that altered miRNAs play important roles in the pathological processes of underlying AD. Another study reported that the expression levels of miRNA in AD patients' brains and cerebrospinal fluid (CSF) were also deregulated; this study also reported the discovery of AD-specific miRNA as potential biomarkers [26]. Compared with the brain tissue, CSF is a feasible clinical sample for diagnostic study. However, CSF is not routinely collected in the evaluation of AD, and there is some risk of infection during lumbar puncture. In comparison, a blood sample is substantially easier to collect, and the identification of biomarker molecules in the blood would be more widely applicable [18]. Some research groups have investigated the levels of A*β*42 and A*β*40 in plasma from AD patients in attempt to develop a specific biomarker; however, there was no significant difference between AD patients and controls for the expression profile of these peptides [18].

Chen et al. and others [9, 11] found numerous stable miRNAs in serum as well as a specific serum miRNA profile that could be used as a biomarker for cancer diagnosis. Many studies were afterward performed to determine the feasibility of diagnosing various cancers using serum miRNAs, including ovarian cancer, colorectal cancer, and non-small cell lung cancer [27–31]. These findings encouraged us to investigate whether serum miRNAs could serve as a noninvasive biomarker for AD despite the fact that the source of serum miRNAs remains unknown. To date, a small number of studies have examined circulating miRNAs in AD patients and have identified several miRNAs that are predictive for AD [14–16]. However, most of these studies only focused on some individual miRNAs, and the sample sizes in those studies were insufficient. Therefore, extensive and genome-wide analyses of the diagnostic value of miRNAs in the circulating blood of AD patients are still needed in large-sample studies. We first employed high-throughput Solexa sequencing to screen the genome-wide expression profile of serum miRNAs in AD. Then, a multiple RT-qPCR assay at the individual level was used to determine the specific miRNA profile of AD. We finally identified four miRNAs, including miR-31, miR-93, miR-143, and miR-146a, that were downregulated in AD compared with the controls.

There was no consensus between this study and previous studies regarding plasma and cerebrospinal fluid (CSF) miRNAs in AD patients [14, 15, 32]. The exact reason for this is unclear; however, we speculate that the inconsistency in miRNA levels may be due to methodology, assay sensitivity, normalization procedures, and sample properties. For example, Tan et al. [32] performed a study on serum miRNA profiles for AD. Their study was conducted using a SYBR primer-based RT-qPCR assay, whereas hydrolysis probe-based RT-qPCR was utilized in our study. Many previous studies demonstrated that hydrolysis probe-based RT-qPCR was more accurate than SYBR primer-based RT-qPCR, and hydrolysis probe-based RT-qPCR has been widely used in the quantification of cell-free miRNA levels. However, normalization procedures represent another important issue in the accurate quantification of RNA levels with RT-qPCR. A common problem in current miRNA research is that no consensus endogenous controls have been established to date. The identified studies used several different genes as their endogenous controls, such as RNU6B, RNU44, RNU48, and miR-16 [33]. However, few reports detailed a robust identification and validation strategy for suitable reference genes for normalization. We attempted several methods of normalizing serum or plasma miRNAs, such as spiking in exogenous miRNA as controls. However, these approaches cannot rule out contamination by preanalytic factors. Therefore, we developed a strategy that normalized the level of circulating miRNAs according to the volume of the serum samples. Previous studies in our laboratory demonstrated that normalization to the volume of the serum samples may represent a feasible method to solve the internal control problem [34, 35]. Unlike our normalization strategy, plasma miRNA expression levels were normalized to spike-in RNA (cel-miR-39) in the study by Tan et al. Furthermore, the diverse, complex molecular events involved in the initiation and development of AD require the functional alteration of not only disease-related genes but also the genes associated with the body's immune responses. Our previous study showed that serum miRNAs are derived not only from circulating blood cells but also from other tissues affected by the disease. The serum miRNA profile in the present study does not have any overlap with that of the previous studies, which found differentially expressed miRNAs in CSF specified from patients with AD. The dysregulation of serum miRNAs may not only be caused by brain lesions but also by changes in the immune system. These miRNAs may participate in disease pathogenesis or progression. Nevertheless, the exact origin of aberrant serum miRNAs is not clear in our study and requires further exploration.

At present, there is no sensitive and specific biomarker for AD. The DSM-IV-TR and NINCDS-AA criteria have been widely used as the “gold standards” for neuropathological disease diagnosis; however, the diagnostic accuracy based on these “gold standards” for AD ranges from 65 to 96%, and the specificity for these diagnostic criteria against other dementias is only 23~88%. Moreover, the real accuracy for the DSM-IV-TR and NINCDS-AA criteria is difficult to assess because the neuropathological standards are not always same across different studies [36]. The four serum miRNAs identified in the present study have a similar sensitivity and specificity using current clinical diagnostic methods. However, we speculate that the panel of these miRNAs has further advantages as a more convenient and specific novel auxiliary diagnosis for the following reasons: (a) they are remarkably stable and reproducible; (b) their concentration profiles are specifically correlated with certain types of neurodegenerative disease; (c) they are easily accessible, are sampled in a relatively noninvasive manner, and are readily detected using RT-qPCR assay, a technique widely used in clinical laboratories; and (d) the cost of the 4-serum biomarkers test is substantially lower than the structural MRI and molecular neuroimaging with PET in a clinical setting and has the potential to be used as a screening marker for high-risk individuals. Moreover, we detected the expression of the four miRNAs in MCI and VD patients using RT-qPCR. MCI patients are considered to have mild cognitive impairment without any obvious symptoms of dementia; these patients generally take care of themselves. However, most of these patients will eventually develop typical AD [5]. Discriminating MCI from control patients will assist in identifying AD patients at an early stage. We can then target therapies that would inhibit the development of the disease and thus avoid deterioration as early as possible. However, dynamic changes in the miRNAs in patients with MCI were not evaluated in the present study due to the relatively small MCI samples, and most of the MCI patients enrolled in this study had not developed AD at data analysis. Thus, the early diagnostic value for our identified serum miRNA panel is difficult to judge. To determine the clinical outcomes of MCI, large sample sizes with MCI and longitudinal followup for several years are needed to discriminate between patients who will and will not develop AD. VD is the second most common type of dementia. Here, we also wanted to determine whether the specific serum miRNAs could differentiate AD from VD, which would be particularly useful in clinical practice. miR-93 and miR-146a were significantly upregulated in MCI compared with the controls and may thus serve as a potential diagnostic molecular biomarker for the development of AD. Furthermore, miR-93 and miR-146a (in addition to miR-31) were also significantly upregulated in VD compared with the controls; thus, miR-31, miR-93, and miR-146a could be specific biomarkers for discriminating AD from VD. However, the four miRNAs cannot discriminate between MCI and VD, which may be because these two diseases share some processes that alter these miRNAs and the nonspecific alterations of the four miRNAs in MCI limit their use as biomarkers for MCI. A systematic study is needed to explore a miRNA serum profile for MCI with high sensitivity and specificity that can accurately discriminate between MCI and VD.

Notably, many miRNAs detected in the serum are upregulated in cancers, whereas most miRNAs are downregulated in Alzheimer's disease. Of the four serum miRNAs selected as biomarkers for AD diagnosis, miR-146a was reported to be brain-enriched and upregulated in the brains of Alzheimer's disease patients compared with the controls. The miR-146a-mediated modulation of CFH gene expression may in part regulate an inflammatory response in AD brains [21]. However, this study found that miR-146a was upregulated in MCI but downregulated in the serum of AD patients. We also noted that miR-31 and miR-93 are increased in MCI but decreased in AD. The exact reason for this alteration is unclear. This inconsistent alteration between MCI and AD may reflect the complex process during disease progression. Notably, miR-31 and miR-93a (in addition to miR-146a) were also related to inflammation, cell apoptosis, and fibrosis [37–42]. Thus, we suspect that the increased miRNA levels in MCI may result from the pathological roles of miRNAs in the initiation process of AD. Additionally, the low serum miRNAs levels in AD may be due to its relatively low neuron density, low inflammation, and high amyloid degeneration. However, we could not determine the exact mechanism in the present study.

In conclusion, we described the expression profile of serum miRNAs in AD and determined that a set of four serum miRNAs could serve as a noninvasive biomarker for diagnosing AD. Moreover, our results will also provide an impetus for future evaluations of the clinical value of serum miRNAs in AD progression, therapeutic efficacy, and prognosis.

## Supplementary Material

Solexa sequencing results of the small RNAs' distribution, the number of known miRNAs and the altered miRNAs in pooled serum from non-dementia controls and AD patients, and RT-qPCR results of the no significantly changed miRNAs between AD serum samples and control samples of training set.

## Figures and Tables

**Table 1:** Demographic and clinical features of the participants whose sera were examined with individual RT-qPCR assays in the training set and the validation set^a^.

Variables	NDC (*n* = 123)	AD (*n* = 127)	MCI (*n* = 30)	VD (*n* = 30)	*P-*value (NDC, AD & NDC, MCI)
Age	79.5 ± 6.8	79.3 ± 8.9	81.1 ± 6.8	82.1 ± 6.8	0.8951^b^; 0.2348^b^
Age group					0.2598^c^; 0.3876^c^
<65 years	4 (3.3%)	8 (6.3%)	2 (6.7%)	1 (3.3%)	
≥65 years	119 (96.7%)	119 (93.7%)	28 (93.3%)	29 (96.7%)	
Gender					0.5412^c^; 0.0895^c^
Male	65 (52.8%)	72 (56.7%)	21 (70.0%)	26 (86.7%)	
Female	58 (47.2%)	55 (43.3%)	9 (30.0%)	4 (13.3%)	
Years of education	9.5 ± 5.4	8.4 ± 5.2	11.4 ± 4.1	10.5 ± 5.3	0.2017^b^; 0.1687^b^
MMSE	27.2 ± 1.3	11.5 ± 7.7	25.6 ± 2.3	8.7 ± 9.0	<0.0001^b^; <0.0001^b^

^a^Data are presented as the means ± SD, ^b^Student's *t*-test, and ^c^two-sided *λ* ^2^ test.

**Table 2:** Differentially expressed miRNAs in AD serum samples compared with control samples in training and validation sets^a^.

miRNA	Training set				Validation set			
	NC (*n* = 48)	AD (*n* = 48)	Fold change	*P-*value^b^	NC (*n* = 75)	AD (*n* = 79)	Fold change	*P-*value^b^
miR-31	10.54 ± 1.19	5.17 ± 0.37	0.491	0.0002	12.39 ± 1.45	5.77 ± 0.84	0.466	<0.0001
miR-93	24.18 ± 2.23	11.72 ± 1.22	0.485	<0.0001	20.63 ± 2.28	10.12 ± 0.98	0.491	<0.0001
miR-143	14.27 ± 1.15	7.03 ± 0.63	0.493	<0.0001	11.98 ± 1.58	5.22 ± 0.78	0.436	<0.0001
miR-146a	73.93 ± 7.16	36.40 ± 2.76	0.492	<0.0001	57.91 ± 6.68	27.26 ± 3.25	0.471	<0.0001

^a^The absolute concentrations of miRNAs are presented as the means ± SEM (fmol/L).

^b^Mann-Whitney unpaired rank-sum test.
