# Supplementary material for: Serum MicroRNA Profiles Serve as Novel Biomarkers for the Diagnosis of Alzheimer's Disease
Source: Dis Markers. 2015 May 20;2015:625659. doi: 10.1155/2015/625659 (PMC4452867; doi:10.1155/2015/625659)
Supplement: Supplementary file 1 — Solexa sequencing results of the small RNAs' distribution, the number of known miRNAs and the altered miRNAs in pooled serum from non-dementia controls and AD patients, and RT-qPCR results of the no significantly changed miRNAs between AD serum samples and control samples of training set. [file 625659.f1.pdf]

Table S1 Distribution of small RNAs in pooled serum from non-dementia controls and AD patients by Solexa sequencing analysis.

	NDC (60 ml)	AD (60 ml)
Total (match genome)	646838	425618
rRNA etc	26.21%	19.82%
miRNA	68.99%	76.15%
repeat	2.19%	1.71%
exon-sense	0.82%	0.84%
exon-antisense	0.05%	0.06%
intron-sense	0.53%	0.50%
intron-antisense	0.39%	0.22%
unannotated	0.81%	0.70%

Table S2 The number of miRNAs in pooled serum from non-dementia controls and AD patients by Solexa sequencing analysis.

Sample	Number of miRNA	Number of miRNA*	Number of hairpin	Number of hairpin-matched reads (unique)	Number of hairpin-matched reads (total)
NDC	239	60	274	1,504	446,261
AD	204	39	242	1,059	324,096

Table S3 Differentially-expressed miRNAs in AD serum samples compared to non-dementia controls determined by Solexa sequencing.

miRNA	Copy number in NDC	Copy number in AD	AD/NDC
hsa-let-7a	11081	5189	0.47
hsa-let-7d	2614	845	0.32
hsa-let-7e	262	112	0.43
hsa-let-7f	1910	746	0.39
hsa-let-7g	526	252	0.48
hsa-let-7i	367	132	0.36
hsa-miR-26a	210	97	0.46
hsa-miR-27a	225	64	0.29
hsa-miR-29a	121	390	3.22
hsa-miR-31	4	61	15.25
hsa-miR-93	124	25	0.20
hsa-miR-103	2190	476	0.22
hsa-miR-107	1414	317	0.22
hsa-miR-106b	2	88	44.0
hsa-miR-199a-3p	734	246	0.33
hsa-miR-148a	49	7	0.14
hsa-miR-7	73	5	0.07
hsa-miR-199b-3p	734	246	0.33
hsa-miR-221	2133	737	0.35
hsa-miR-222	8788	3784	0.43
hsa-miR-223	334	102	0.30
hsa-miR-23b	78	15	0.20
hsa-miR-27b	180	68	0.38
hsa-miR-122	48207	137395	2.85
hsa-miR-143	54	24	0.44
hsa-miR-191	712	300	0.42
hsa-miR-146a	275	103	0.38
hsa-miR-193a-5p	112	291	2.60
hsa-miR-148b	52	2	0.03
hsa-miR-324-3p	79	36	0.45
hsa-miR-339-3p	109	41	0.37
hsa-miR-483-5p	205	502	2.45
hsa-miR-574-5p	33	66	2.00
hsa-miR-886-5p	429	191	0.45
hsa-miR-874	28	73	2.60
hsa-miR-885-3p	8	64	8.05
hsa-miR-1308	48	352	7.34
hsa-miR-1307	1446	515	0.36

Table S4 MiRNAs showing no significantly alteration in AD serum samples compared to control samples in training set by RT-qPCR.[a]

miRNA	NC (n=48)	AD (n=48)	Fold change	*p*-value[b]
miR-122	16.52±3.20	15.56±2.06	0.94	0.7993
miR-23a	0.97±0.15	0.66±0.08	0.69	0.0677
miR-103	2.66±0.46	4.04±0.74	1.52	0.1118
miR-193a-5p	3.62±1.09	2.00±0.32	0.55	0.1504
miR-874	0.65±0.11	0.54±0.07	0.84	0.4100
miR-23b	1.06±0.29	1.27±0.18	1.19	0.5471
miR-221	1.08±0.20	1.11±0.16	1.03	0.9167
miR-199a-3p	0.16±0.02	0.18±0.03	1.18	0.4820
miR-7	1.32±0.26	0.80±0.12	0.60	0.0652
miR-148a	1.33±0.21	0.78±0.13	0.59	0.0257
miR-191	12.29±1.52	6.78±0.88	0.55	0.0020
miR-885-3p	10.42±1.28	9.69±1.41	0.93	0.6979

[a]The absolute concentrations of miRNAs are presented as mean ± SEM (fmol/L).

[b] Mann-Whitney unpaired test for rank sum.
